# Effects and Mechanisms of Radiofrequency Ablation of Renal Sympathetic Nerve on Anti-Hypertension in Canine

**DOI:** 10.5935/abc.20170014

**Published:** 2017-03

**Authors:** Wei Chen, Xiaohong Tang, Xiaofang Yang, Chunyan Weng, Kan Yang, Juan Wen, Hong Liu, Yang Wu

**Affiliations:** Department of Cardiology - the Third Xiangya Hospital - Central South University, Changsha - Hunan

**Keywords:** Sympatectomy, Hypertension, Renal Insufficiency, Radio Waves, Dogs

## Abstract

**Background:**

Radiofrequency ablation of renal sympathetic nerve (RDN) shows effective BP reduction in hypertensive patients while the specific mechanisms remain unclear.

**Objective:**

We hypothesized that abnormal levels of norepinephrine (NE) and changes in NE-related enzymes and angiotensinconverting enzyme 2 (ACE2), angiotensin (Ang)-(1-7) and Mas receptor mediate the anti-hypertensive effects of RDN.

**Methods:**

Mean values of systolic blood pressure (SBP), diastolic blood pressure (DBP) and mean arterial pressure (MAP) were assessed at baseline and follow-up. Plasma and renal norepinephrine (NE) concentrations were determined using highperformance liquid chromatography with electrochemical detection, and levels of NE-related enzyme and ACE2-Ang(1-7)- Mas were measured using real time PCR, Western blot and immunohistochemistry or Elisa in a hypertensive canine model fed with high-fat diet and treated with RDN. The parameters were also determined in a sham group treated with renal arteriography and a control group fed with normal diet.

**Results:**

RDN decreased SBP, DBP, MAP, plasma and renal NE. Compared with the sham group, renal tyrosine hydroxylase (TH) expression was lower and renalase expression was higher in the RDN group. Compared with the control group, renal TH and catechol-o-methyl transferase (COMT) were higher and renalase was lower in the sham group. Moreover, renal ACE2, Ang-(1-7) and Mas levels of the RDN group were higher than those of the sham group, which were lower than those of the control group.

**Conclusion:**

RDN shows anti-hypertensive effect with reduced NE and activation of ACE2-Ang(1-7)-Mas, indicating that it may contribute to the anti-hypertensive effect of RDN.

## Introduction

Hypertension is the leading cause of cardiovascular diseases worldwide,^1^ resulting in an estimated 7.6 million deaths annually.^2^ Globally, 40.8% of the population is affected by hypertension, with an awareness rate of 46.5% and a control rate of 32.5%.^3^ The control of hypertension is a challenge due to the side effects, low compliance and limited efficacy of anti-hypertensive drugs.

The anti-hypertensive effects of radiofrequency ablation of renal sympathetic nerve (RDN) were first reported by Henry Krum in 2009.^4^ The Symplicity HTN-1^5^ and Symplicity HTN-2^6^ trials showed profound anti-hypertensive effects during a follow-up period of 36 months. A meta-analysis confirmed the effectiveness of RDN therapy for resistant hypertension,^7^ and was found superior to maximal medical therapy in lowering blood pressure (BP).^8^ However, the Symplicity HTN-3 study did not show effective BP reduction in resistant hypertensive patients,^9^ indicating that only a minority of patients was eligible for RDN. 

Since the specific anti-hypertensive mechanisms of RDN are not clear, norepinephrine (NE) concentrations are an index of sympathetic neural activity in humans, which is positively correlated to BP. RDN may decrease NE that contributes to low BP, although the effect of RDN on NE is inconsistent.^10,11^ The inconsistent NE levels after RDN may be caused by tyrosine hydroxylase (TH), renalase, catechol-o-methyl transferase (COMT) and norepinephrine transporter (NET) activity, which are the enzymes associated with the synthesis and metabolism of NE. On the other hand, the angiotensin-converting enzyme 2 (ACE2)/ angiotensin (Ang)-(1-7)/Mas axis constitutes an alternative to the renin - angiotensin system (RAS) and represents an intrinsic mechanism to induce vaso-protective actions by counter regulating the ACE/AngII/AT1R axis, thus inducing many beneficial effects on cardiovascular diseases (CVDs). It is inversely related to BP^12^ and exhibits cardiovascular and renal protection. Therefore, we aim to determine the effect of RDN on renal NE and changes in NE-related enzymes (TH, renalase, COMT and NET). Additionally, we investigated the levels of renal ACE2 - Ang (1-7) - Mas axis after RDN and discussed the potential anti-hypertensive mechanisms of RDN.

## Methods

### Animal preparation

All procedures on the use and care of animals was approved by the Ethical Committee of Central South University. Beagle dogs (n=28, 10 to 12 months of age, weighing 11 ~ 12kg) were randomly divided into a hypertensive model group (n = 22) and a control group (n = 6). Throughout the study, dogs were fed high-fat diet (lard 0.3 ~ 0.4kg/day was added to 250g/day regular diet) in the model group and regular diet (250g/day including 23% protein, 11% fat, 4.9% fiber, water 10%, 1-3% calcium, 0.8% phosphorus, 0.29% methionine, vitamin A 11000IU / kg, vitamin D3 1000 IU / kg and vitamin E 500 IU / kg) in the control group. After 3 months of high-fat diet, 20 dogs achieved an approximate 50% increase in body weight, and the fat intake was reduced to a maintenance level. This model of canine obesity following a high-fat diet closely mimics the cardiovascular, renal, hormonal, and metabolic changes observed in obese human subjects. The hypertensive group was divided into a surgery group (n = 10) and a sham surgery group (n = 10). Three dogs were excluded for the following reasons: retroperitoneal hematoma caused by femoral artery puncture (n=1) and death due to anesthesia (n=2). The surgery group (n=9) was treated with radiofrequency ablation of the renal sympathetic nerve, and the sham surgery (n=8) and control groups (n=6) were treated with renal arteriography. Six months after RDN, we sacrificed the beagle dogs under deep anesthesia by intramuscular injection of pentobarbital sodium (30-35mg/kg).

### Radiofrequency ablation of the renal sympathetic nerve

Surgery was performed at room temperature, with prior fasting for 24 hours, and after anesthetizing dogs with an intramuscular injection of sodium pentobarbital (30-35mg / kg). After successful anesthesia, the dogs were placed in the supine position on the operating table, followed by routine disinfection of the right femoral artery. A catheter was inserted through the femoral artery to monitor BP and renal arteriography. The radiofrequency ablation catheter was inserted through the femoral artery into the renal artery and connected to a radiofrequency ablation device (IBI, St. Jude Medical, Inc., St. Paul, MN, USA). Three to four ablation sites were selected from each site and a spiral shape local ablation was performed (5F IBI radiofrequency ablation catheter; St. Jude Medical). Each spot was ablated for 120 sec, with a power limit of 8 W, until the tumor temperature reached 55°C. Renal arteriography was performed immediately after the surgery, pressure was applied at the puncture point for 15 - 30 min, bandaged and fixed.

### Analytic methods

Body weight was determined by electronic scales. SBP, DBP and MAP were measured by BP-2010E (Softron, China), a tail arterial blood pressure measuring instrument. Plasma concentration and renal tissue levels of NE were measured by high-performance liquid chromatography with electrochemical detection (HPLC). Renal ACE2 and Mas mRNA levels were measured by real time PCR. Renal Ang (1-7) (Cusabio, China) was estimated by ELISA. TH, renalase, COMT, NET, ACE2 and Mas protein expression levels in renal tissue were measured by Western blot and immunohistochemistry.

### HPLC

NE in acidified release medium, perfusate, and superperfusate samples was identified and quantified by HPLC. The system consists of a Varian Pro-Star solvent delivery system and a model 9090 autosampler (Varian, Walnut Creek, CA), coupled to a C18 column and an ESA Coulochem II detector. Separations were performed isocratically using a filtered and degassed mobile phase, consisting of 12% methanol, 0.1 M sodium phosphate, 0.2 mM sodium octyl sulfate, and 0.1 mM EDTA, adjusted to pH 2.8 with phosphoric acid. The high-pressure liquid chromatography system is coupled to a computer, where the chromatograms were recorded and analyzed using the Varian Star workstation software.

### Western blot

Frozen tissues were lysed with cell lysis buffer containing protease inhibitor. The protein concentration of each specimen was measured based on the Bradford method utilizing the Bio-Rad Protein Assay Kit (Bio-Rad Laboratories, Hercules, CA, USA) with bovine serum albumin (BSA) as the standard. After the protein denaturing procedure with loading buffer, each sample (50 *µ*g) was resolved on 8-12% SDS-polyacrylamide gel electrophoresis (PAGE) gel (Bio-Rad Laboratories) at room temperature and transferred onto a polyvinylidene fluoride membrane at 4°C. After blocking in 5% non-fatty milk for 1 hour at room temperature, it was incubated overnight at 4°C with polyclonal rabbit anti-TH antibody (1:500 Abcam, USA), polyclonal goat anti-renalase antibody (1:500, biorbyt, USA), polyclonal goat anti-COMT antibody (1:500, LifeSpan BioSciences, USA), polyclonal rabbit anti-NET antibody (1:500, Abcam, USA), polyclonal goat anti- ACE2 antibody (1:200, Santa Cruz, USA) and polyclonal rabbit anti-Mas antibody (1:200, Santa Cruz, USA) with β-actin (1 : 1000, Abcam, USA) as the positive control. The HRP-conjugated rabbit anti-goat (1 : 2000) or goat anti-rabbit (1 : 3000) secondary antibody was added and the membranes were then incubated for 1 hour at room temperature. After washing, signals were visualized by luminol reagents (Bio-Rad Laboratories) and the densitometry of each blot was analyzed with the latest version of Scion Image 4.0.3.2.

### Immunohistochemistry

The staining procedure was performed on paraffin-embedded renal tissue sections (5*µ*m). Antigen was retrieved from all the sections by boiling with sodium citrate buffer (pH 6) and incubating with polyclonal rabbit anti-TH antibody (1:150 Abcam, USA), polyclonal goat anti- renalase antibody (1:150, biorbyt, USA), polyclonal goat anti-COMT antibody (1:150, LifeSpan BioSciences, USA), polyclonal rabbit anti-NET antibody (1:150, Abcam, USA), polyclonal goat anti- ACE2 antibody (1:100, Santa Cruz, USA) and polyclonal rabbit anti-Mas antibody (1:100, Santa Cruz, USA) overnight at 4°C. After staining, all specimens were dehydrated and sealed for microscopic observation. Protein occurrence and distribution in antibody-stained tissue sections were observed using the Nikon eclipse E400 microscope and Digital HyperHAD Color Video Camera (Sony) using the Easy Image Analysis software (NIS-Elements BR v3.0) for evaluation of immunostaining.

### Statistical analyses

Results were expressed as means ± standard error of the means, and all data passed a normality test. Comparisons between the hypertensive model group and control groups were done using the unpaired Student's *t-*test assuming unequal variance, whereas within 3 groups analysis was performed using one-way ANOVA with a Neuman-Keuls post hoc analysis. Paired Student's *t-*test was used to compare before and after establishing the hypertensive model, and before and after RDN. Linear correlation was used to evaluate the association between SBP and level of the above mentioned factors. All data were analyzed by SPSS 22.0. P < 0.05 was considered significant.

## Results

### Canine model of hypertension and response to RDN

**Figure 1:**
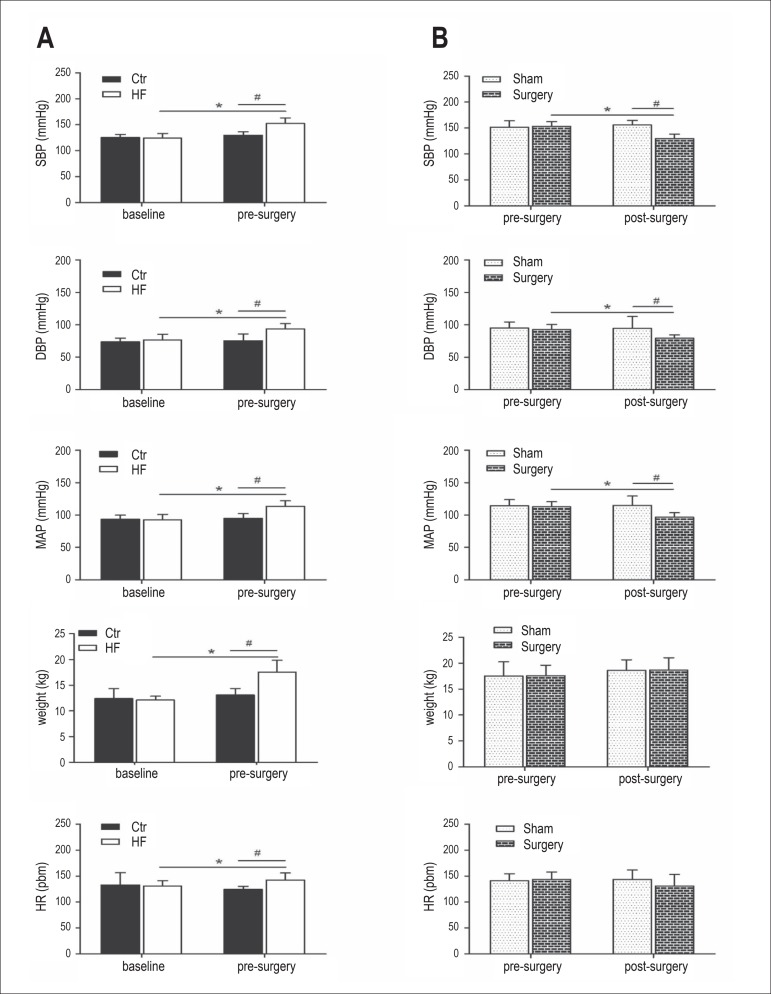
Effects of high-fat diet on body weight, HR and BP (SBP, DBP and MAP) of beagle dogs (A). Body weight, HR and BP (SBP, DBP and MAP) responses to RDN (B). Values are mean ± SEM.*p < 0.05 versus baseline, # p < 0.05 versus control group in figure 2A. *p < 0.05 versus pre-surgery of surgery group, # p < 0.05 versus sham surgery group in figure 2B. SBP: Systolic blood pressure; DBP: Diastolic blood pressure; MAP: Mean arterial pressure; HR: Heart rate.

**Figure 2:**
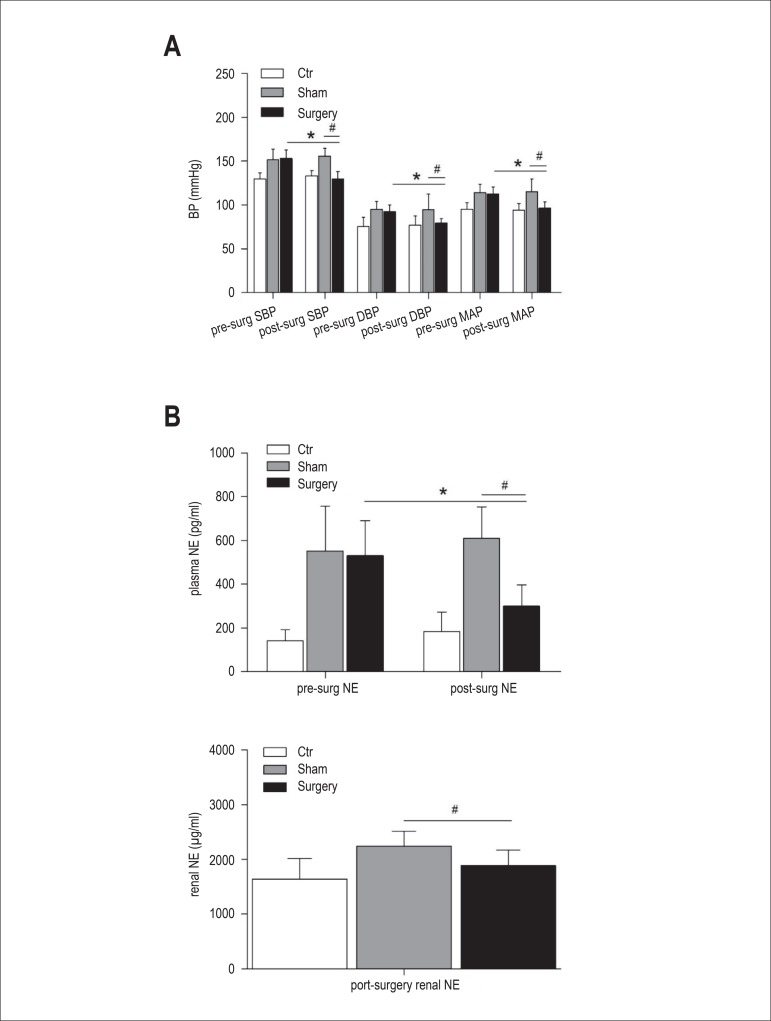
BP (SBP, DBP and MAP)(A) and NE (plasma and renal) (B) in response to RDN. Values are mean ± SEM.*p < 0.05 versus pre-surgery of surgery group, # p < 0.05 versus sham surgery group in figure 2. BP: blood pressure; SBP: Systolic blood pressure; DBP: Diastolic blood pressure; MAP: Mean arterial pressur; NE: norepinephrine.

After 3 months on high-fat diet, there was a marked increase in body weight, HR, SBP, DBP and MAP of the hypertensive group (*p < 0.05 vs. baseline, # p < 0.05 vs. control group). SBP, DBP and MAP of hypertensive group increased by approximately 28±10mmHg, 17±8mmHg and 21±8mmHg, respectively, along with the target weight gain of 45.2%. Moreover, plasma NE in the hypertensive group was remarkably increased after 3 months of high-fat diet (*p < 0.05 vs. baseline, # p < 0.05 vs. control group). (Figure 1). SBP, DBP and MAP of the surgery group dramatically decreased by approximately 24±9mmHg, 13±6mmHg and 16±7mmHg 6 months after surgery, respectively. When compared with the sham surgery group, SBP, DBP and MAP of the surgery group also declined significantly. (Figure 2A) Six months after RDN, plasma NE of the surgery group was significantly reduced (*p < 0.05 vs. pre-surgery of surgery group, # p < 0.05 vs. sham surgery group). In addition, renal NE in the surgery group was also lower than the in the sham surgery group (p < 0.05). (Figure 2B)

### Levels of renal TH, renalase, COMT and NET response to RDN

**Figure 3:**
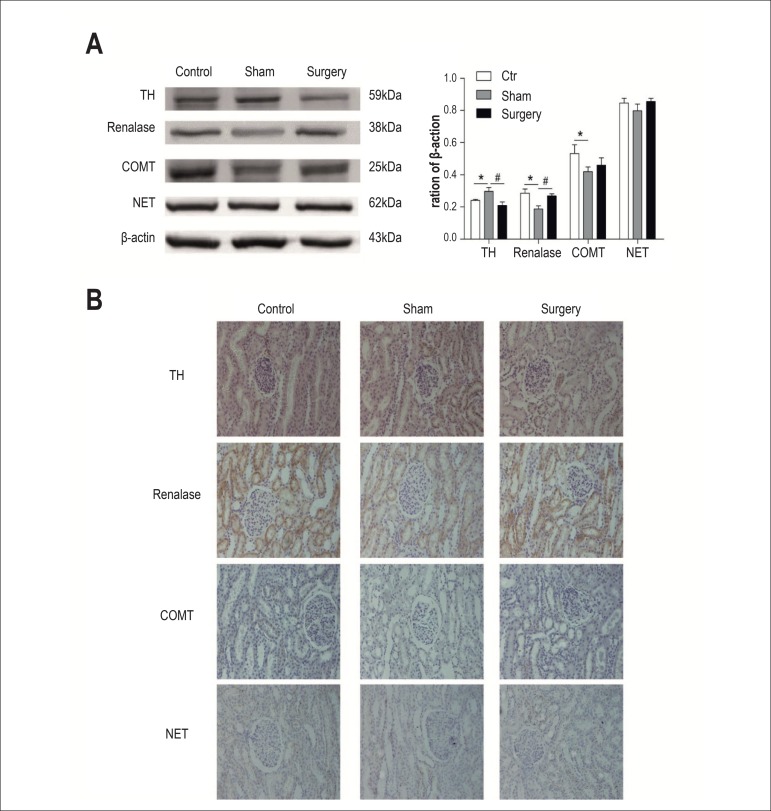
Effects of RDN on renal TH, renalase, COMT and NET protein expression of beagle dogs. Values are mean±SEM. * p < 0.05 versus control group, # p < 0.05 versus sham surgery group. TH: tyrosine hydroxylase ; COMT: catechol-o-methyl transferase; NET: norepinephrine transporter.

Six months after surgery, renal TH protein expression in the surgery group was lower than in the sham surgery group (p < 0.05). TH immunohistochemical staining (brown) was located in the cytoplasm of renal tubules in beagle dogs (Figure 3A). Kidney level of renalase in the surgery group was significantly higher than in the sham surgery group (p < 0.05). Immunohistochemical results showed that renalase protein was expressed in the cytoplasm of renal tubular epithelial cells (Figure 3B). The renal COMT protein expression in the sham surgery group was lower than in the control group (p < 0.05). In the immunohistochemical study, COMT was located in the cytoplasm of renal tubules in beagle dogs (Figure 3C). NET was located in the cytoplasm of renal tubules in beagle dogs. However, there was no statistical difference of the renal NET among the 3 groups (Figure 3D).

### Levels of renal ACE2, Ang-(1-7) and Mas response to RDN

**Figure 4:**
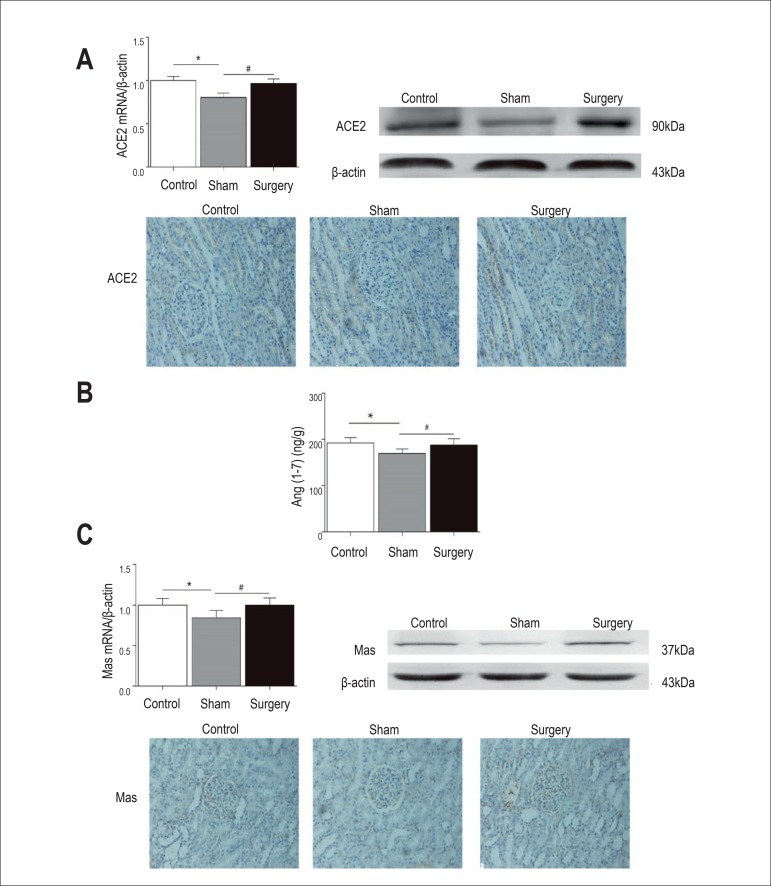
Effects of RDN on renal ACE2 mRNA and protein expression (A), Ang-(1-7) concentration (B) and Mas mRNA and protein expression(C) of beagle dogs. Values are mean±SEM. * p < 0.05 versus control group, # p < 0.05 versus sham surgery group. ACE2 mRNA: angiotensin-converting enzyme 2.

Renal ACE2 mRNA and protein expression in the surgery group were significantly higher than in the sham surgery group (p < 0.05). Immunohistochemical staining (brown) of ACE2 was located in the cytoplasm and membrane of renal tubules in beagle dogs. Six months after surgery, values with positive area density in the surgery group were significantly stronger than in the sham group (p < 0.05) (Figure 4A). Similar to ACE2, renal tissue Ang-(1-7) concentration in the sham surgery group was the lowest, dramatically lower than in the surgery group and control group (p < 0.05) (Figure 4B). And the level of renal Mas was also higher in the surgery group and control group than in the sham surgery group. Immunohistochemical staining (brown) of Mas was located in the renal glomeruli and proximal tubule cell cytoplasm and cell membrane in beagle dogs (Figure 4C).

## Discussion

In our study, obesity-related hypertension, induced by high-fat diet, was associated with increase in body weight, HR, SBP, DBP and MAP, and this was in agreement with the results of previous studies.^13^ RDN effectively reduced blood pressure for SBP, DBP and MAP by approximately 24±9mmHg, 13±6mmHg and 16±7mmHg in the surgery group after 6 months of surgery, respectively. SBP, DBP and MAP of the surgery group also declined significantly when compared with the sham surgery group, which was similar to findings of previous clinical studies^5,6^ and animal experiment^14^. The Symplicity HTN-1 trial enrolled 153 resistant hypertensive patients, of whom 111 agreed to the 36-month follow-up. SBP (-32.0mmHg) and DBP(-14.4mmHg) decreased significantly.^5^ The Symplicity HTN-2 trial randomized 106 subjects with resistant hypertension, whose SBP and DBP were reduced by 33 mmHg and 14 mmHg at 36 months, respectively.^6^ However, the Symplicity HTN-3 study did not show effective BP reduction in resistant hypertensive patients,^9^ indicating that only a minority of patients was eligible for RDN. 

NE is an important indicator of sympathetic neural activity, and is elevated in diseases with high sympathetic activity. Tiroch et al.^10^ found that RDN resulted in a marked decrease of the NE spillover rate, while Machino et al.^11^ observed no significant difference after RDN of systemic NE in spontaneously hypertensive rats (SHR). We observed that plasma NE increased significantly in an obesity-related hypertensive canine model, and decreased nearly 44% with RDN. Furthermore, RDN was effective in reducing renal NE concentration, consistent with the results of Rimoldi et al.^15^ As reported, RDN was associated with additional benefits^16^ and improvement of the cardiac and renal functions.^17,18^ It was also proposed as a promising treatment in diseases with sympathetic overactivation.^19,20^ In this study, we determined the antihypertensive value of RDN using an obesity-related hypertensive canine model with high NE levels.

Renal TH level was also increased in obesity-related hypertension in this study. In addition, RDN decreased the renal TH protein expression in the hypertensive model, which is substantially similar to previous studies. Down-regulation of TH in the adrenal medulla of SHR was accompanied by a potent decrease of NE and SBP,^21^ suggesting that RDN may influence NE concentrations by affecting the renal TH level. On the other hand, renalase was remarkably lower in the obesity-related hypertensive canine model in this study, suggesting an inverse relationship with hypertension similar to findings of previous studies.^22^ Desir GV showed that recombinant renalase *in vitro* or *in vivo* lowers blood pressure by degrading plasma adrenaline, with its antihypertensive effect directly related to its enzymatic activity.^23^ SHR plasma and renal renalase levels were profoundly increased after RDN compared with the baseline, sham and control groups, with MAP significantly decreased,^24^ which is consistent with this study, suggesting that RDN may lower the NE concentrations by elevating the renal renalase expression. COMT and NET are major enzymes involved in degrading catecholamines, which is inversely related to hypertension. As shown in our study, renal COMT level profoundly lowered obesity-related hypertension compared with the control group, while RDN was ineffective against the expression of renal COMT and NET. As they are mainly expressed in nerve endings, it suggested that renal COMT and NET may have had no significant change after RDN.

The ACE2-Ang-(1-7)-Mas axis is involved in hypertension. Transgenic mice overexpressing growth hormone showed increased SBP, a high degree of both cardiac and renal fibrosis and a markedly decreased level of ACE2-Ang-(1-7)-Mas, and Ang-(1-7) administration reduced SBP.^25^ Activation of the ACE2-Ang-(1-7)-Mas pathway reduces oxygen-glucose deprivation-induced tissue swelling, ROS production, and cell death in mouse brain associated with angiotensin II overproduction.^26^ Consistent with the previous study, in addition to the reduction of ACE2, we also observed that renal Ang-(1-7) concentration and Mas mRNA and protein expression decreased in obesity-related hypertension. We first found that RDN increased renal ACE2-Ang-(1-7)-Mas axis in an obesity-related hypertensive canine model.

RDN shows anti-hypertensive effect with reduced NE and activation of ACE2-Ang-(1-7)-Mas, indicating that this may contribute to the anti-hypertensive effect of RDN. However, the relationship between these two pathways was not clear in this study. Ang-(1-7) elicits a facilitatory presynaptic effect on peripheral noradrenergic neurotransmission,^27,28^ and is inhibitory at the central nervous system through the Mas receptor.^29,30^ It is suggested that ACE2-Ang(1-7)-Mas may decrease the concentration of NE to achieve the anti-hypertensive effect, however, this needs to be confirmed by further studies.

Nevertheless, this study has limitations as follows. Firstly, the number of dogs is small, and this may lead to a bias result. Secondly, changes of NE, NE-related enzymes and ACE2-Ang-(1-7)-Mas were detected during the procedure, while the relationship between these changes was unclear. The variation of renal TH, renalase and ACE2-Ang-(1-7)-Mas may have affected the level of NE to contribute to the anti-hypertensive effect of RDN. These limitations should be addressed in further studies to clarify the possible mechanisms of RDN, thus contributing to develop and improve this new treatment method.

## Conclusions

The initial question that motivated our study was to determine whether NE and ACE2-Ang-(1-7)-Mas would prove to participate in the antihypertensive effect of RDN. Our study confirmed that RDN shows an antihypertensive effect with reduced plasma and renal NE, which may be related to the decrease of TH and increase of renalase in the kidney. Furthermore, RDN activates the ACE2-Ang-(1-7)-Mas pathway and this may contribute to the antihypertensive effect of RDN. Although the application of RDN is not clear because of its varying effectiveness, our data suggested that it may be an excellent choice in obesity-related hypertension patients with high levels of NE and over-activation of the renin-angiotensin system.
